# Immunological Role of *TP53* Somatic Mutation Classification in Human Cancers

**DOI:** 10.1155/2023/1904309

**Published:** 2023-02-13

**Authors:** Jianfei Fang, Ying Yang, Lina Xie, Wenjuan Yin

**Affiliations:** ^1^Department of Pathology, The Cancer Hospital of the University of Chinese Academy of Sciences (Zhejiang Cancer Hospital), Institute of Basic Medicine and Cancer(IBMC), Chinese Academy of Sciences, Hangzhou, Zhejiang 310022, China; ^2^The Second Clinical Medical College, Zhejiang Chinese Medicine University, Hangzhou, Zhejiang 310053, China

## Abstract

**Background:**

*TP53* is a very common tumor suppressor gene and has implicated in various cancers. A systematic immunological analysis of *TP53* somatic mutation classification in multiple cancers is still lacking.

**Methods:**

To assess the immunological value of *TP53* somatic mutation classification in various cancers, we integrated a series of bioinformatics methods to analyze the role of *TP53* gene across the public databases, such as UCSC Xena, Cancer Cell Line Encyclopedia (CCLE), Ensembl, and Genotype−Tissue Expression (GTEx).

**Results:**

The results revealed that the *TP53* expression level had significant difference in tumor tissues and normal tissues, and it had a high expression level in most malignant tumors. Moreover, the missense mutation is the most common type of *TP53* mutation in most cancers. In addition, the Cox proportional hazards model analysis and Kaplan−Meier (KM) survival analysis demonstrated that the *TP53* expression is a high-risk factor in brain lower-grade glioma (LGG), prostate adenocarcinoma (PRAD), and uterine carcinosarcoma (UCS), which is opposite in uterine corpus endometrial carcinoma (UCEC). Besides, compared to the *TP53* nontruncating mutation classification samples, we found that *TP53* truncating mutation samples had lower *TP53* expression levels in certain types of cancer. Notably, *TP53* was associated with the mismatch repair (MMR) gene in some cancers which contained truncating or nontruncating mutation. Based on the classification of truncating or nontruncating mutation, we also discovered that *TP53* expression was positively or negatively correlated with the immune score, stromal score, and the levels of immune cell infiltration in different cancers.

**Conclusions:**

Our research reveals an overarching landscape of immunological value on *TP53* status in various malignant tumors. According to our results, we demonstrate that *TP53* also plays an immunological role in various cancers.

## 1. Introduction

Cancer is a malignant disease with a high mortality rate. Up till now, there is no effective treatment to absolutely cure cancer patients which most likely predicts poor quality of life [1]. However, immunotherapy has been reported to achieve great improvement and regarded as one of the major breakthroughs in cancer treatments [2–4]. It is feasible to seek out potential immunotherapy biomarker through the utility of databases which contained sequencing data.

*TP53* is a well-studied tumor suppressor gene and is always the hotspot of tumor research. Nowadays, studies on *TP53* function have concentrated on the correlation between variation pattern of *TP53* and prognosis [5–7]. *TP53* has a prominent role in preventing tumor development and maintaining genomic stability (GS) [8, 9]. The cancer-associated function of the p53 protein depends on its five function domains, including transactivation domain, proline rich domain, DNA-binding domain, oligomerization domain, and carboxy-terminal regulatory domain [10, 11]. In various cancers, *TP53* was explicitly linked to cancer development and prognosis [12–14], but these conclusions were still controversial [15]. The most widely accepted concept is that the nontruncated mutation commonly induces high expression of *TP53* [13]. Previous studies have demonstrated that *TP53* mutations are associated with their expression, cancer prognosis [13], and immune − related research [16]. Integrated studies combined with these features with classification of *TP53* somatic mutation are few.

Our work makes it possible to explore the association between *TP53* mutation classification and immunological function by multiple databases, such as UCSC Xena, CCLE, Genotype – Tissue Expression (GTEx), and Ensembl. This study may provides new perspective on the relationship between gene variation classification and microenvironment for researchers. We present the following article in accordance with the MDAR reporting checklist.

## 2. Methods

### 2.1. *TP53* Gene Expression Data

The expression data across 33 types of cancers which contains 11057 samples were downloaded from UCSC Xena. The expression data across 31 health tissues which contains 7858 samples and 28 tumor cell lines were downloaded from GTEx and CCLE. Expression data were extracted using in-house Perl scripts and further analysis using R software (version 4.1.2, R Foundation for Statistical Computing, Vienna, Austria).

### 2.2. *TP53* Variation and Classification Criteria

In this part, the varscan2 variation files with *TP53* gene form UCSC Xena (accessed 18 February 2022) were used for analyzing base alterations. According to the mutation classification method in previous studies [13, 15], we distinguished between truncating (including nonsense mutation, frameshift mutation, and splice site mutation) and nontruncating (including missense mutation, in-frame deletion mutation, and in-frame insertion mutation) *TP53* mutation.

### 2.3. Correlation between *TP53* and Prognosis

We obtained relevant clinical data from the UCSC Xena. Overall survival (OS) data of patients were used to estimate the prognosis status of different *TP53* classification. The patients were divided into two sections by the mean value of *TP53* expression as a cutoff value [17]. The KM method and log-rank test were used for survival analysis. Moreover, Cox proportional hazards model analysis was performed to analyze the hazard ratio among different types of cancer.

### 2.4. Correlation between *TP53* and Mismatch Repair Genes

We calculated the Pearson correlation coefficient to estimate the relationship between *TP53* and mismatch repair (MMR) genes, including *MLH1*, *MSH2*, *MSH6,* and *PMS2*. *P* < 0.05 was considered for the statistical significance.

### 2.5. Correlation between *TP53* and Immunity

We used the ESTIMATE algorithm to infer the degree of tumor infiltration [18, 19]. Moreover, the CIBERSORT method was further used for exploring the correlation between *TP53* and immunity [20]. The annotation file with Ensembl gene converted to gene symbol was downloaded by Ensembl database.

### 2.6. Correlation between *TP53* and Biological Function

It is convenient to investigate the function of *TP53* in various cancers using GSEA package. All analyses were completed by R software (version 4.1.2, R Foundation for Statistical Computing, Vienna, Austria).

### 2.7. Statistical Analysis

The gene expression values were converted to fragments per kilobase per million for subsequent analysis. *P* < 0.05 was considered for the statistical significance. All statistical analysis was processed by the R software (version 4.1.2, R Foundation for Statistical Computing, Vienna, Austria).

## 3. Results

### 3.1. The Landscapes of *TP53* Expression across Multiple Datasets

**Figure 1:**
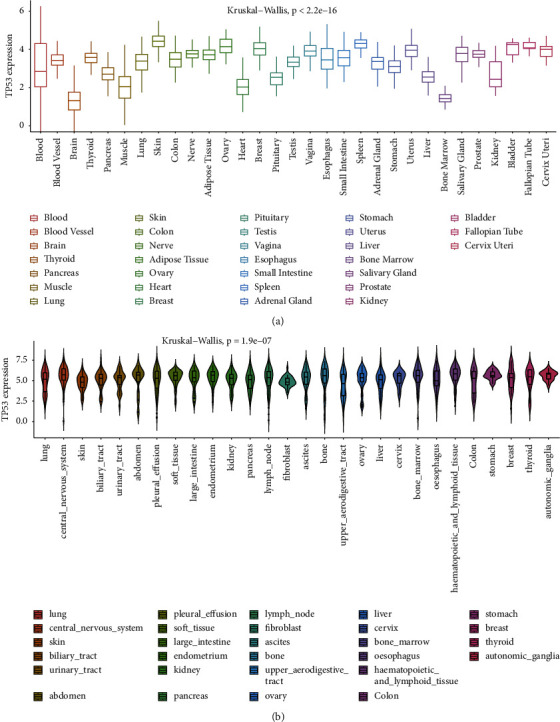
The expression levels of *TP53* in GTEx datasets and CCLE datasets. (a) *TP53* expression in normal tissues using GTEx datasets. (b) *TP53* expression in tumor cell lines using CCLE datasets.

In various normal samples, we found significant difference of *TP53* expression, as shown in Figure 1(a) (*P* < 2.2*e* − 16). The skin tissue expresses *TP53* on the highest level and the brain tissue expressed the lowest. We further extracted *TP53* expression values from cancer cell lines using CCLE datasets, and the consequence was presented in Figure 1(b), displaying the analogous difference in cell lines (*P*=1.9*e* − 07).

**Figure 2:**
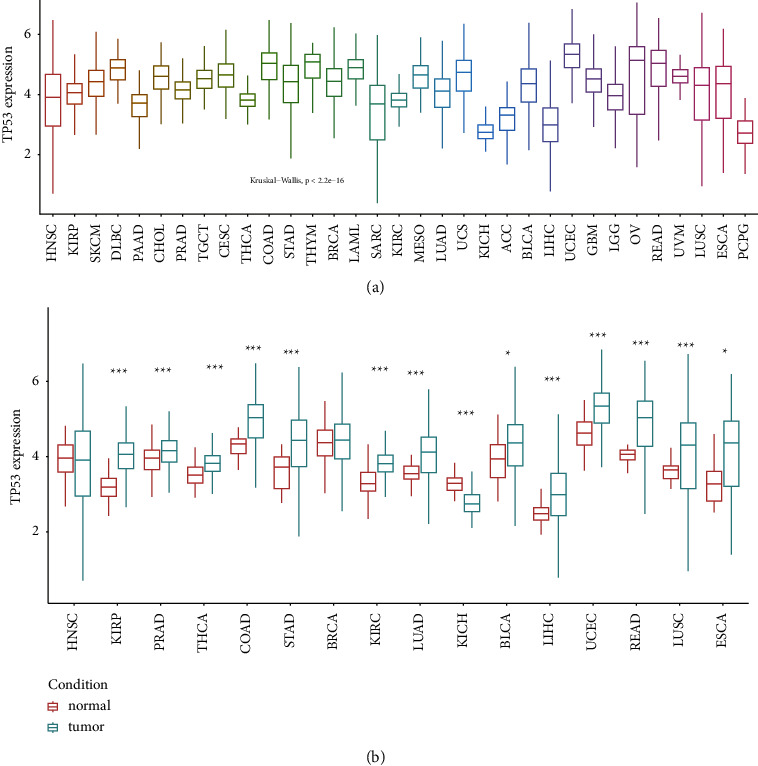
Expression level of *TP53* in UCSC Xena datasets. (a) *TP53* expression in tumor samples. (b) *TP53* expression in tumor-normal samples. ^*∗*^indicates *P* < 0.05, ^*∗∗*^indicates *p* < 0.01, ^*∗∗∗*^indicates *P* < 0.001.

In addition, we discovered the similar significance of *TP53* expression in 33 cancer types (*P* < 2.2*e* − 16). As shown in Figure 2(a), pheochromocytoma and paraganglioma (PCPG) and kidney chromophobe (KICH) are on the lowest level while UCEC is on the highest level.

To further investigate the difference among tumor-normal samples, we analyzed the UCSC Xena data. In view of the fact that some types of cancers included only a few normal samples (for instance, less than 10 or no normal samples datasets), 16 types of cancer were retained for further analysis (Figure 2(b)). Compared with normal samples, we observed higher expression of *TP53* in most cancer types, for instance, lung squamous cell carcinoma (LUSC, *P*=0.0001894), rectum adenocarcinoma (READ, *P*=0.007547), stomach adenocarcinoma (STAD, *P*=1.229*e* − 06), colon adenocarcinoma (COAD, *P*=4.4*e* − 09), kidney renal papillary cell carcinoma (KIRP, *P*=3.848*e* − 12), uterine corpus endometrial carcinoma (UCEC, *P*=1.655*e* − 08), prostate adenocarcinoma (PRAD, *P*=0.0003865), bladder urothelial carcinoma (BLCA, *P*=0.0321), lung adenocarcinoma (LUAD, *P*=3.794*e* − 09), liver hepatocellular carcinoma (LIHC, *P*=5.799*e* − 06), kidney renal clear cell carcinoma (KIRC, *P* < 2.2*e* − 16), thyroid cancer (THCA, *P*=4.995*e* − 12), and esophageal cancer (ESCA, *P*=0.01165). In contrast, *TP53* expression was downregulated in kidney chromophobe (KICH, *P*=1.145*e* − 07). Moreover, there were no significant difference in *TP53* expression levels in breast invasive cancer (BRCA, *P*=0.2613) and head and neck squamous cell carcinoma (HNSC, *P*=0.9583).

### 3.2. *TP53* Mutation in Pan-Cancer Cohorts

**Figure 3:**
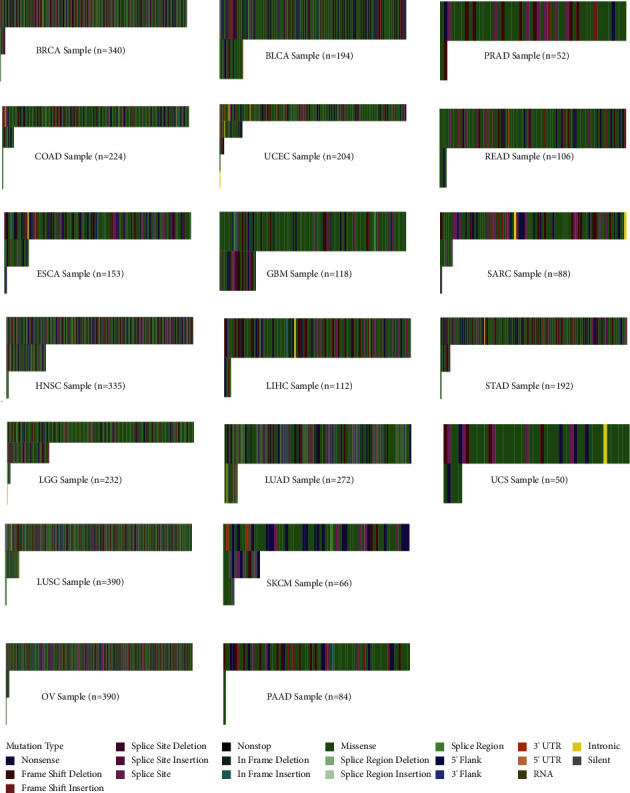
Landscape of somatic mutations of *TP53* gene in pan-cancer using the UCSC Xena cohort.

After eliminating those cancers with the small number samples (for instance, cancer types with less than thirty samples were excluded), 19 cancer types were screened for depicting the *TP53* mutation types in pan-cancer, and the landscape was exhibited in Figure 3. Consistent with the previous studies [13, 21], we found that missense mutations accounted for the most of *TP53* variants in 19 types of cancer. Next, nonsense mutation ranked second.

### 3.3. Prognostic Impact of *TP53* across *TP53*-Mut Cancers

**Figure 4:**
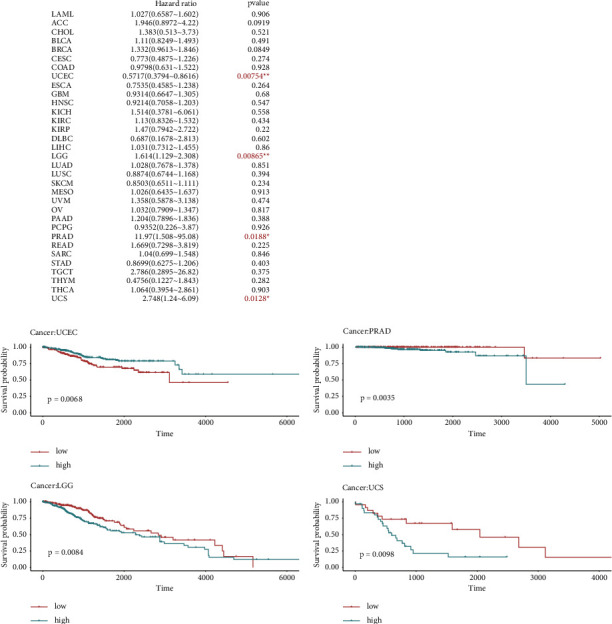
Association between *TP53* expression and overall survival time across *TP53*-mut cancer types. ^*∗*^indicates *P* < 0.05, ^*∗∗*^indicates *P* < 0.01, ^*∗∗∗*^indicates *P* < 0.001.

In Figure 4, forest plots showed that *TP53* was high-risk gene in LGG (hazard ratio = 1.614), PRAD (hazard ratio = 11.97), and UCS (hazard ratio = 2.748), while it was a low-risk gene in UCEC (hazard ratio = 0.5717). Furthermore, we carried out prognostic impact of *TP53* expression using mean value as criteria and revealed the significant difference between high and low expression groups among LGG (*P*=0.0084), PRAD (*P*=0.0035), UCS (*P*=0.0098), and UCEC (*P*=0.0068).

### 3.4. *TP53* Mutation Classification and Survival

**Figure 5:**
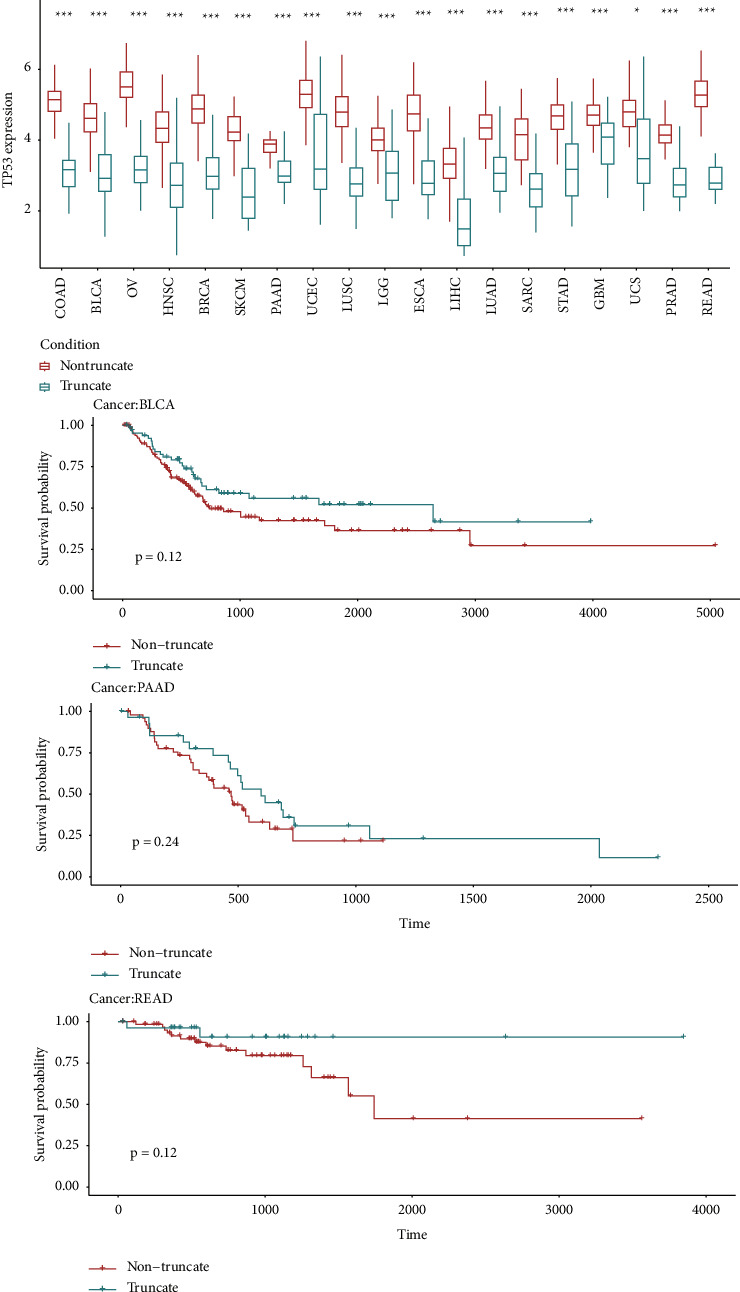
Association between *TP53* mutation classification and overall survival time in *TP53*-mut cancers. ^*∗*^indicates *P* < 0.05, ^*∗∗*^indicates *P* < 0.01, ^*∗∗∗*^indicates *P* < 0.001.

We distinguished *TP53* mutations into truncating and nontruncating classes to observe their effects on *TP53* expression. In line with the earlier conclusion, the results (see Figure 5) showed that *TP53* expression levels were upregulated in truncating mutation relative to nontruncating patients [15].

Furthermore, we performed survival analysis for the sake of the evaluation of *TP53* mutation's prognostic value. Unfortunately, no significance difference was detected between *TP53* mutation types and overall survival time in any type of cancer (Figure 5). However, KM (Kaplan−Meier) results indicated a clear trend that individuals with truncating mutation had longer survival time in specific cancers, such as READ, BLCA, and PAAD.

### 3.5. Correlation between *TP53* and Mismatch Repair Genes

**Figure 6:**
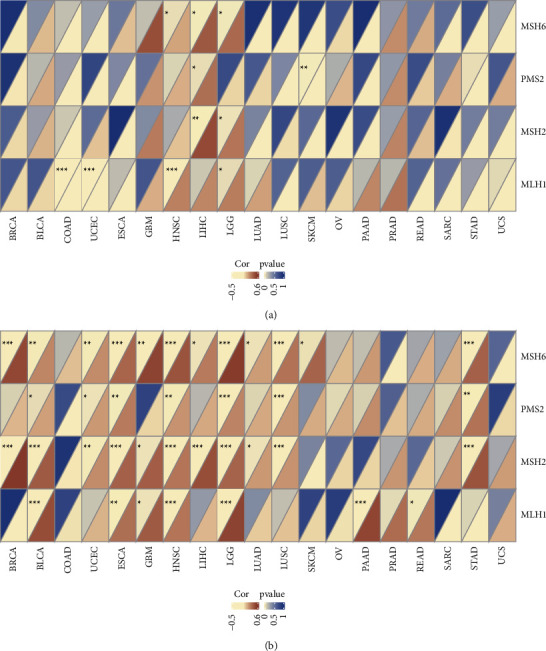
Association between *TP53* expression and MMR genes in 19 *TP53*-mut cancers. (a) The relationship between *TP53* expression and MMR genes in truncating *TP53*-mut cancers. (b) The relationship between *TP53* expression and MMR genes in nontruncating *TP53*-mut cancers. For each cell, the top left triangle represents the *P* value and the bottom right triangle represents the correlation coefficient. ^*∗*^indicates *P* < 0.05, ^*∗∗*^indicates *P* < 0.01, ^*∗∗∗*^indicates *P* < 0.001.

Mismatch Repair (MMR) is a typical DNA repair mechanism [22]. Ectopic expression of MMR genes might induce the high frequency of somatic mutations [23, 24]. In truncating *TP53*-mut cancer types (see Figure 6(a)), we examined that MMR genes had significant positive correlation with HNSC (*MLH1* correlation coefficient/*P*-value = 0.27/0.00096; *MSH6* correlation coefficient/*P*-value = 0.17/0.04), LIHC (*MSH2* correlation coefficient/*P*-value = 0.47/0.003; *PMS2* correlation coefficient/*p*-value = 0.33/0.04; *MSH6* correlation coefficient/*P*-value = 0.41/0.0104), and LGG (*MLH1* correlation coefficient/*P*-value = 0.28/0.047; *MSH2* correlation coefficient/*P*-value = 0.31/0.02; *MSH6* correlation coefficient/*P*-value = 0.35/0.012). In contrast, negative correlation with *TP53* expression was discovered in four cancers, including COAD (*MLH1* correlation coefficient/*P*-value = −0.42/0.0009), UCEC (*MLH1* correlation coefficient/*P*-value = −0.47/0.0005), SKCM (*PMS2* correlation coefficient/*P*-value = −0.5/0.0097), and STAD (*PMS2* correlation coefficient/*P*-value = −0.25/0.04).

In nontruncating *TP53*-mut cancer types (Figure 6(b)), we also explored the positive correlation across multiple cancers. Interestingly, compared with different *TP53* nontruncating classification, there was opposite correlations in UCEC (*MSH6* correlation coefficient/*P*-value = 0.24/0.005), SKCM (*MSH6* correlation coefficient/*P*-value = 0.37/0.03), and STAD (*MSH2* correlation coefficient/*P*-value = 0.43/3.267*e* − 05; *PMS2* correlation coefficient/*P*-value = 0.32/0.003; *MSH6* correlation coefficient/*P*-value = 0.38/0.0003).

### 3.6. Correlations between *TP53* and Immunity

Tumor microenvironment (TME) played an important role during neoplasm occurrence and progression [16, 25, 26]. In this study, we calculate the stromal score, immune score, and estimate score in 19 *TP53*-mut cancers using the ESTIMATE method. As shown in Table 1, in *TP53* truncating mutation cancers, such as BRCA, HNSC, LIHC, LUAD, LUSC, SKCM, PAAD, SARC, and STAD, *TP53* was significantly positively correlated with the immune score, as well as stromal score and estimate score, while it was negatively correlated with UCS. Conversely, in nontruncating mutation cancers (for instance, BRCA, GBM, OV, and PRAD), *TP53* was significantly negatively associated with the estimated TME scores, while LIHC and PAAD had the opposite consequences.

Subsequently, we investigated the immune cell infiltration levels among *TP53*-mut caners. The results implied the diverse significant correlation between levels of immune cell infiltration and *TP53* expression among *TP53* truncating or nontruncating mutant cancers. Significant correlation is screened and presented in Table 2.

### 3.7. Correlation between *TP53* and Biological Function

We carried out a thorough inspection of Gene Set Enrichment Analysis (GSEA) to investigate the relationship between *TP53* and biological function in*TP53*-mut tumor tissues, and the results are shown in Supplementary Figures (Available here).

**Figure 7:**
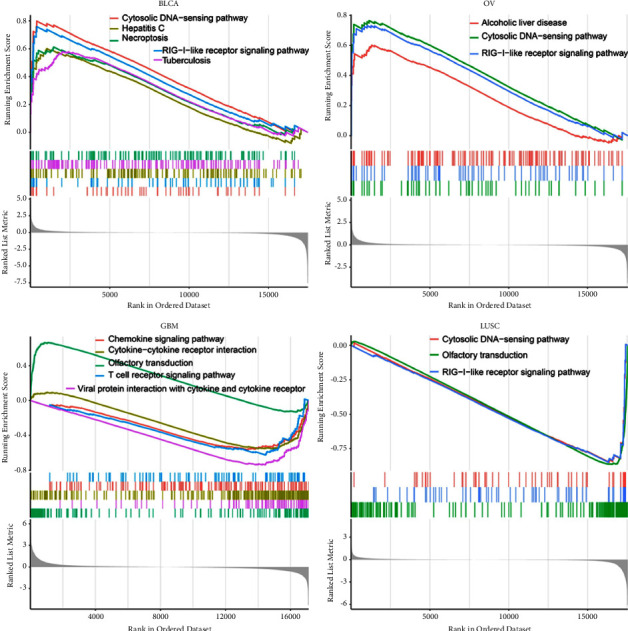
KEGG analysis of *TP53* in BLCA, OV, GBM, and LUSC. Peaks on the upward curve indicate positive regulation and peaks on the download curve indicate negative regulation.

**Figure 8:**
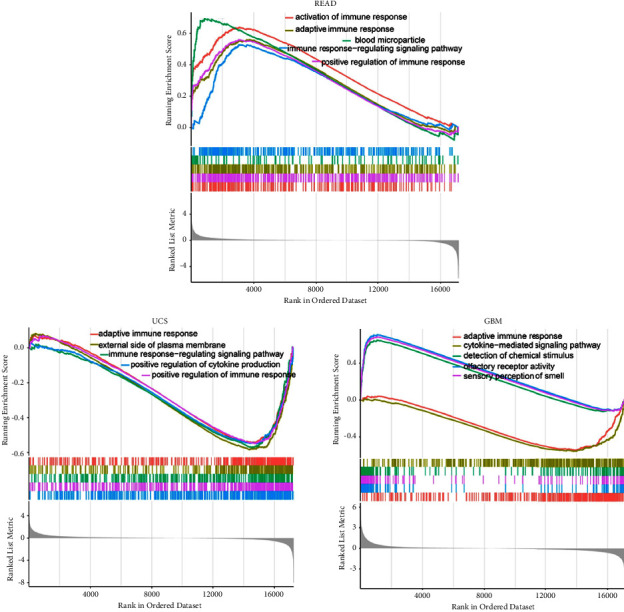
GO analysis of *TP53* in READ, GBM, and UCS. Peaks on the upward curve indicate positive regulation and peaks on the download curve indicate negative regulation.

In *TP53* truncating vs nontruncating mutation cancers, the KEGG data indicated that *TP53* positively regulated RIG-I-like receptor signal pathway [27] and cytosolic DNA-sensing pathway [28, 29] in BLCA and OV. In contrast, *TP53* was predicted to be a negative regulator of the T cell receptor signaling pathway [30], cytosolic DNA-sensing pathway and RIG-I-like receptor signal pathway in GBM, and/or LUSC (Figure 7). In GBM and UCS, the GO data showed that *TP53* expression was negatively correlated with adaptive immune response, immune response − regulating signal pathway and had a positive regulation with immune response. In READ, *TP53* expression exhibited the opposite effect (see Figure 8).

## 4. Discussion

In this article, the expression level of *TP53* gene was diverse in tumor or normal tissues. *TP53* expression was higher in most cancers than in normal tissues except KICH.

Besides, we analyzed the variation of *TP53* in 19 cancers and discovered that missense mutation was the dominant subtype, which is consistent with the previous conclusion [13]. At the same time, the evidences showed that patients with the higher expression of *TP53* had a worse survival in UCS, LGG, and PRAD. On the contrary, in UCEC, the higher expression of *TP53* gene was linked to better survival.

In previous studies, the prognosis roles of *TP53* mutation were controversial [15]. Meanwhile, the expression level of *TP53* is often related to the mutation types [13]. In order to examine the impact of *TP53* mutations on prognosis, *TP53* mutations were divided into truncating and nontruncating mutation groups referring to the published reliable classification method [13]. Our results demonstrated that the patients with truncating mutations presented lower *TP53* expression. Besides, Kaplan−Meier analysis showed a clear trend that individuals with truncating *TP53* mutation had longer survival time in BLCA (*P*-value = 0.12), PAAD (*P*-value = 0.24), and READ (*P*-value = 0.12), consistent with the results of previous published literature [13].

Dan et al. [22] clarified that the abnormal expression of mismatch repaired genes induced the increased frequency of somatic mutation. In colorectal cancer, Perez et al. [31] indicated that mismatch-repaired deficiency can induce *TP53* mutation. Fang et al. [32] reported that *TP53* defection and mismatch-repaired deficiency commonly occurred in early carcinosarcoma. The correlation between *TP53* mutation classification and the expression of the MMR genes was analyzed. In the truncating mutation group, *TP53* expression is positively correlated with MMR genes expression in LGG, LIHC, and HNSC, while negative correlation was found in other four cancers, including COAD, SKCM, UCEC, and STAD. In contrast, in the nontruncating mutation group, we detected the positive correlation in most cancers. Compared to *TP53* nontruncating classification, there were opposite correlations in UCEC, SKCM, and STAD.

We further explored the relationship between *TP53* mutation classification and tumor microenvironment. According to the ESTIMATE algorithm [18, 19], we calculated the stromal score, immune score, and estimate score. In the *TP53* truncating mutation group, *TP53* is significantly positively correlated with immune score, as well as stromal score and estimate score in specific cancers, such as BRCA, HNSC, LIHC, LUAD, LUSC, SKCM, PAAD, SARC, and STAD. Whereas, it is negatively correlated in UCS. Conversely, in the nontruncating mutation group, *TP53* is significantly negatively correlated with stromal/estimate/immune score in BRCA, GBM, OV, and PRAD, while LIHC and PAAD had opposite results.

Correlation between the degree of immune cell infiltration and *TP53* expression was estimated further. In truncating or nontruncating mutation samples, obvious associations between the previous two factors were shown in most cancers. Finally, GSEA results indicated that *TP53* (truncating or nontruncating) was involved in some immune−related function and pathways.

Some limitations of this paper include the limited databases and scarce experimental verification. In the future, we will adopt more valuable databases and experimental results to confirm and improve our work.

## 5. Conclusions

In conclusion, this might be the first comprehensive and systematic research to evaluate the immune − related mechanisms of *TP53* mutation classification in different cancer. According to our results, *TP53* is related to immunological function based on different mutation classification in various cancers. It is worth mentioning that these findings might extend better understanding of *TP53* gene underlying the mechanism in the immune system.

## Figures and Tables

**Table 1:** Correlation between *TP53* expression and tumor microenvironment in 19 *TP53*-mut cancers.

Cancer types	*TP53* classification	Cor/*P*-value		
		Stromal score	Immune score	Estimate score
BRCA	Truncating	0.32/0.0003^*∗∗∗*^	0.3/0.0006^*∗∗∗*^	0.35/5.607*e* − 05^*∗∗∗*^
	Nontruncating	−0.4/7.994*e* − 09^*∗∗∗*^	0.07/0.34	−0.2/0.02^*∗*^

BLCA	Truncating	0.11/0.37	0.13/0.28	0.13/0.28
	Nontruncating	−0.05/0.56	−0.2/0.07	−0.1/0.2

COAD	Truncating	0.09/0.51	0.16/0.22	0.13/0.31
	Nontruncating	−0.1/0.22	−0.12/0.14	−0.1/0.16

UCEC	Truncating	0.13/0.35	0.03/0.85	0.08/0.6
	Nontruncating	−0.1/0.21	−0.16/0.053	−0.15/0.07

ESCA	Truncating	0.08/0.58	−0.08/0.59	0.0009/0.995
	Nontruncating	−0.01/0.92	−0.21/0.06	−0.11/0.31

GBM	Truncating	0.07/0.84	0.16/0.64	0.13/0.71
	Nontruncating	−0.3/0.07	−0.32/0.053	−0.33/0.04^*∗*^

HNSC	Truncating	0.3/0.0002^*∗∗∗*^	0.3/0.0002^*∗∗∗*^	0.32/7.295*e* − 05^*∗∗∗*^
	Nontruncating	0.1/0.18	0.04/0.56	0.08/0.29

LIHC	Truncating	0.45/0.004^*∗∗*^	0.42/0.007^*∗∗*^	0.47/0.002^*∗∗*^
	Nontruncating	0.15/0.21	0.24/0.046^*∗*^	0.22/0.07

LGG	Truncating	0.14/0.32	−0.14/0.32	−0.01/0.94
	Nontruncating	−0.09/0.22	0.03/0.72	−0.02/0.81

LUAD	Truncating	0.27/0.009^*∗∗*^	0.3/0.005^*∗∗*^	0.31/0.003^*∗∗*^
	Nontruncating	−0.03/0.72	−0.09/0.3	−0.07/0.43

LUSC	Truncating	0.28/0.002^*∗∗*^	0.25/0.004^*∗∗*^	0.28/0.0014^*∗∗*^
	Nontruncating	−0.05/0.4	−0.07/0.29	−0.07/0.31

SKCM	Truncating	0.7/6.962*e* − 05^*∗∗∗*^	0.8/1.285*e* − 06^*∗∗∗*^	0.81/5.301*e* − 07^*∗∗∗*^
	Nontruncating	−0.09/0.62	−0.15/0.39	−0.13/0.45

OV	Truncating	0.15/0.16	−0.006/0.95	0.08/0.47
	Nontruncating	−0.17/0.044^*∗*^	−0.13/0.11	−0.16/0.054

PAAD	Truncating	0.32/0.09	0.47/0.0105^*∗*^	0.43/0.02^*∗*^
	Nontruncating	0.23/0.11	0.4/0.004^*∗∗*^	0.34/0.02^*∗*^

PRAD	Truncating	−0.08/0.75	0.08/0.76	−0.005/0.98
	Nontruncating	−0.36/0.04^*∗*^	−0.24/0.18	−0.32/0.07

READ	Truncating	0.36/0.06	0.26/0.18	0.34/0.08
	Nontruncating	−0.08/0.53	0.05/0.68	−0.02/0.87

SARC	Truncating	0.52/0.002^*∗∗*^	0.24/0.17	0.4/0.02^*∗*^
	Nontruncating	0.08/0.56	0.008/0.96	0.04/0.8

STAD	Truncating	0.46/0.0001^*∗∗∗*^	0.27/0.03^*∗*^	0.4/0.0008^*∗∗∗*^
	Nontruncating	−0.01/0.92	−0.06/0.61	−0.04/0.74

UCS	Truncating	−0.61/0.04^*∗*^	−0.13/0.7	−0.43/0.17
	Nontruncating	−0.07/0.7	0.2/0.24	0.09/0.62

^*∗*^indicates *P* < 0.05; ^*∗∗*^indicates *P* < 0.01; ^*∗∗∗*^indicates *P* < 0.001.

**Table 2:** Significant correlation between *TP53* expression and immune cell infiltration in 19 *TP53*-mut cancers.

Cell types	*TP53* classification in 19 *TP53*-mut cancers (cor/*P*-value)	
	Truncating	Non-truncating
Naive B cells	UCS: 0.65/0.02^*∗*^	BLCA: 0.22/0.02^*∗*^
		LGG: −0.23/0.002^*∗∗*^
		LUAD: 0.18/0.03^*∗*^

Memory B cells	PAAD: 0.38/0.045^*∗*^	NA

Plasma cells	NA	LGG: −0.17/0.02^*∗*^

CD8 T cells	UCEC: 0.41/0.003^*∗∗*^	BRCA: 0.22/0.002^*∗∗*^
	SKCM: 0.45/0.02^*∗*^	LIHC: 0.25/0.04^*∗*^
		LUAD: 0.18/0.04^*∗*^
		LUSC: 0.16/0.012^*∗*^

Naive CD4 T cells	HNSC: −0.2/0.04^*∗*^	LGG: −0.18/0.02^*∗*^
	OV: 0.37/0.0002^*∗∗∗*^	READ: −0.3/0.02^*∗*^

Resting CD4 memory T cells	UCEC: −0.37/0.007^*∗∗*^	BRCA: −0.2/0.007^*∗∗*^
		PAAD: −0.4/0.009^*∗∗*^
		PRAD: −0.4/0.04^*∗*^

Activated CD4 memory T cells	NA	BRCA: 0.3/1.872*e* − 05^*∗∗∗*^
		GBM: −0.4/0.02^*∗*^

Follicular T helper cells	UCEC: 0.34/0.014^*∗*^	BRCA: 0.3/1.876*e* − 05^*∗∗∗*^
	OV: −0.24/0.02^*∗*^	LGG: 0.16/0.03^*∗*^
	STAD: 0.3/0.013^*∗*^	

Regulatory T cells	LUSC: 0.22/0.011^*∗*^	LUSC: 0.14/0.03^*∗*^
	SKCM: 0.44/0.03^*∗*^	

Gamma delta T cells	OV: 0.36/0.0003^*∗∗∗*^	GBM: −0.33/0.04^*∗*^
		LUAD: −0.22/0.009^*∗∗*^
		PAAD: 0.65/3.765*e* − 07^*∗∗∗*^
		PRAD: 0.4/0.02^*∗*^

Resting NK cells	BLCA: 0.31/0.013^*∗*^	BRCA: −0.2/0.02^*∗*^
	SKCM: −0.4/0.04^*∗*^	READ: −0.3/0.03^*∗*^
	OV: 0.34/0.0007^*∗∗∗*^	SARC: 0.36/0.011^*∗*^

Activated NK cells	NA	COAD: 0.22/0.008^*∗∗*^
		UCEC: −0.18/0.03^*∗*^
		GBM: 0.49/0.002^*∗∗*^
		LGG: 0.23/0.002^*∗∗*^
		PRAD: 0.46/0.007^*∗∗*^

M0 macrophages	ESCA: 0.4/0.004^*∗∗*^	UCEC: 0.21/0.014^*∗*^
	LUSC: 0.22/0.02^*∗*^	OV: 0.25/0.002^*∗∗*^
	OV: 0.24/0.02^*∗*^	
M1 macrophages	NA	BRCA: 0.14/0.045^*∗*^
		BLCA: −0.2/0.047^*∗*^
		PAAD: 0.3/0.03^*∗*^
		PRAD: −0.43/0.013^*∗*^

M2 macrophages	BRCA: −0.25/0.005^*∗∗*^	BRCA: −0.4/8.285*e* − 09^*∗∗∗*^
	LGG: −0.31/0.03^*∗*^	HNSC: 0.21/0.006^*∗∗*^
	UCS: −0.6/0.046^*∗*^	LUAD: −0.2/0.04^*∗*^

Resting dendritic cells	SKCM: 0.39/0.047^*∗*^	NA

Activated dendritic cells	BRCA: −0.23/0.008^*∗∗*^	BRCA: 0.15/0.04^*∗*^
	OV: −0.2/0.03^*∗*^	HNSC: −0.2/0.014^*∗*^
	PRAD: 0.6/0.008^*∗∗*^	

Resting mast cells	COAD: 0.4/0.002^*∗∗*^	BRCA: −0.4/3.896*e* − 08^*∗∗∗*^
		LIHC: −0.25/0.04^*∗*^
		SARC: −0.35/0.014^*∗*^

Activated mast cells	HNSC: −0.2/0.004^*∗∗*^	BRCA: 0.21/0.003^*∗∗*^
	LIHC: 0.37/0.02^*∗*^	HNSC: −0.3/0.0001^*∗∗∗*^
		LUAD: −0.2/0.03^*∗*^
		LUSC: −0.1/0.047^*∗*^
		UCS: 0.44/0.008^*∗∗*^

Eosinophils	NA	BRCA: 0.14/0.04^*∗*^
		COAD: −0.2/0.04^*∗*^
		LGG: 0.18/0.02^*∗*^
		SKCM: −0.5/0.004^*∗∗*^

Neutrophils	HNSC: −0.2/0.02^*∗*^	COAD: −0.22/0.006^*∗∗*^
	STAD: −0.26/0.03^*∗*^	HNSC: −0.2/0.008^*∗∗*^
		LUAD: −0.2/0.012^*∗*^
		LUSC: −0.22/0.0006^*∗∗∗*^

Monocytes	NA	COAD: −0.2/0.04^*∗*^
		GBM: −0.36/0.03^*∗*^
		OV: −0.25/0.002^*∗∗*^
		STAD: −0.27/0.011^*∗*^

^*∗*^indicates *P* < 0.05; ^*∗∗*^indicates *P* < 0.01; ^*∗∗∗*^indicates *P* < 0.001. NA indicates no statistical correlation.

## Data Availability

The datasets generated and analyzed during the current study are available in the three public repositories, namely, UCSC Xena (https://xena.ucsc.edu/), CCLE (https://portals.broadinstitute.org/ccle/), and GTEx (https://commonfund.nih.gov/GTEx).

## Supplementary Materials

Supplementary Materials Figure S1: KEGG pathway analysis of *TP53* in multiple cancers. Peaks on the upward curve indicate positive regulation and peaks on the download curve indicate negative regulation. Figure S2: GO analysis of *TP53* in multiple cancers. Peaks on the upward curve indicate positive regulation and peaks on the download curve indicate negative regulation.
