# Supplementary material for: Immunological Role of TP53 Somatic Mutation Classification in Human Cancers
Source: J Oncol. 2023 Feb 13;2023:1904309. doi: 10.1155/2023/1904309 (PMC9940963; doi:10.1155/2023/1904309)
Supplement: Supplementary Materials — Figure S1: KEGG pathway analysis of TP53 in multiple cancers. Peaks on the upward curve indicate positive regulation and peaks on the download curve indicate negative regulation. Figure S2: GO analysis of TP53 in multiple cancers. Peaks on the upward curve indicate positive regulation and peaks on the download curve indicate negative regulation. [file 1904309.f1.zip › Supplementary Figures Results of GSEA.pdf]

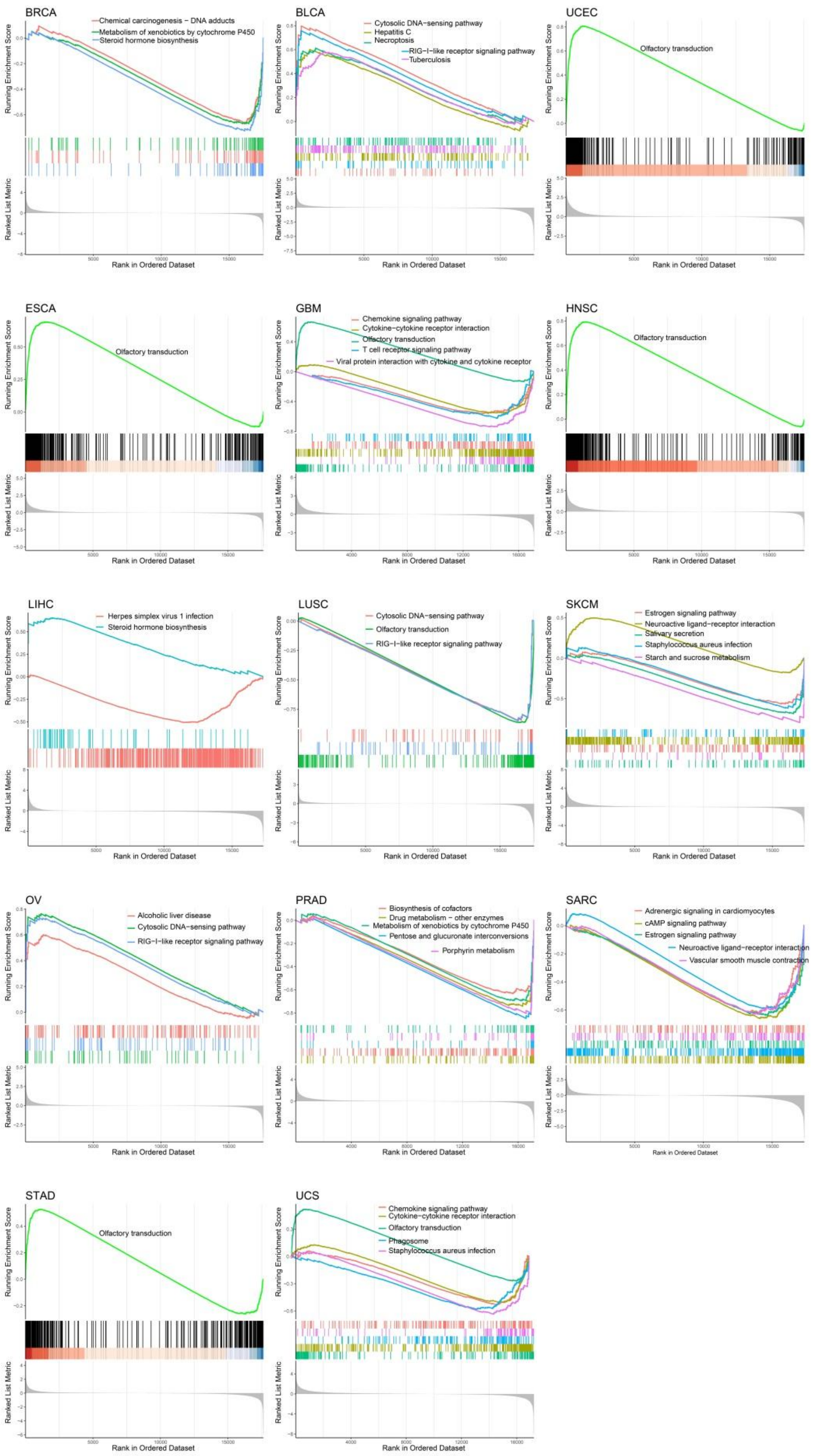

Figure S1 KEGG pathway analysis of *TP53* in multiple cancers. Peaks on the upward curve indicate positive regulation and peaks on the download curve indicate negative regulation.

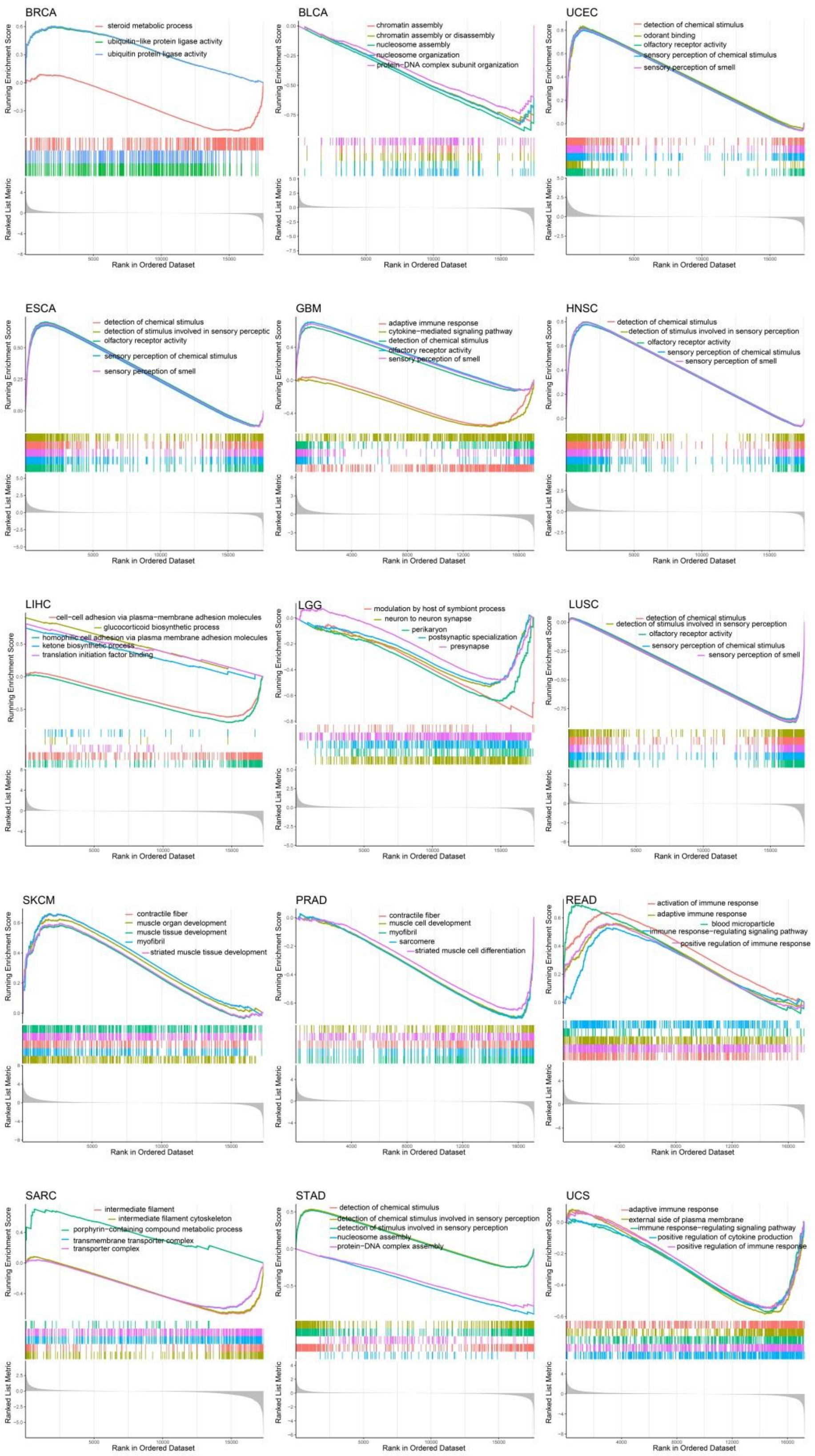

Figure S2 GO analysis of *TP53* in multiple cancers. Peaks on the upward curve indicate positive regulation and peaks on the download curve indicate negative regulation.
